# Incidence and predictors of under-five mortality in East Africa using multilevel Weibull regression modeling

**DOI:** 10.1186/s13690-021-00727-9

**Published:** 2021-11-12

**Authors:** Getayeneh Antehunegn Tesema, Achamyeleh Birhanu Teshale, Zemenu Tadesse Tessema

**Affiliations:** Department of Epidemiology and Biostatistics, institute of public health, College of medicine and health science, University of Gondar, Gondar, Ethiopia

**Keywords:** Under-five mortality, East Africa, Multilevel Weibull regression model

## Abstract

**Background:**

In 2017, an estimated 5.3 million under-five children died annually in Sub-Saharan African countries, more than half of those deaths occurred in East Africa. Though East African countries share the huge burden of global under-five mortality, there is limited evidence on the incidence and predictors of under-five mortality. Therefore, this study investigated the incidence and predictors of under-five mortality in East Africa.

**Methods:**

A community-based cross-sectional study was done based on the Demographic and Health Survey (DHS) data of 12 East African countries conducted from 2008 to 2019. A total weighted sample of 138,803 live births within 5 years preceding the survey were included for analysis. The Kaplan-Meier curve and Log-rank test were done to assess the children’s survival experience across variable categories. The Global Schoenfeld residual test was employed for checking Proportional Hazard (PH) assumptions and it was violated (*p*-value< 0.05). Considering the hierarchical nature of DHS data, multilevel parametric survival models were fitted. Model comparison was made by AIC, deviance, and shape of the hazard function. Variables with a *p*-value of less than 0.2 in the bi-variable analysis were considered for the multivariable analysis. In the multilevel Weibull regression analysis, the Adjusted Hazard Ratio (AHR) with 95% Confidence Interval (CI) was reported to declare the significant predictors of under-five mortality.

**Results:**

Under-five mortality rate in East Africa was 51.318 (95% CI: 51.311, 51.323) per 1000 live births. Babies born to mothers attained secondary education and above (AHR = 0.83, 95% CI: 0.75, 0.91), being 2nd - 4th birth order (AHR = 0.62, 95% CI: 0.56, 0.67), ≥ 5th birth order (AHR = 0.68, 95% CI: 0.61, 0.76), health facility delivery (AHR = 0.87, 95% CI: 0.82, 0.93), 1–3 ANC visit (AHR = 0.61, 95% CI: 0.54, 0.68), births interval of 24–48 months (AHR = 0.53, 95% CI: 0.50, 0.57), wanted pregnancy (AHR = 0.72, 95% CI: 0.68, 0.76), middle wealth status (AHR = 0.90, 95% CI: 0.83, 0.97), and richest wealth status (AHR = 0.81, 95% CI:0.73, 0.90) were significantly associated with lower hazards of under-five mortality. Whereas, advanced maternal age (≥35 years) (AHR = 1.13, 95% CI: 1.04, 1.24),, babies born to household who did not have media exposure (AHR = 1.13, 95% CI: 1.07, 1.20), twin births (AHR = 3.81, 95% CI: 3.52, 4.12), being male child (AHR = 1.27, 95%CI: 1.21, 1.33), small birth size at birth (AHR = 1.73, 95% CI: 1.63, 1.84), and large size at birth (AHR = 1.11, 95% CI: 1.04, 1.11) were significantly associated with higher hazards of under-five mortality.

**Conclusion:**

Under-five mortality is a major public health concern in East African countries. Health facility delivery, ANC visit, higher wealth status, adequate birth spacing, wanted pregnancy, and maternal education were significantly correlated with a lower risk of under-5 mortality. Whereas, higher birth order, small or large size at birth, male birth, twin birth, advanced maternal age and mothers who didn’t have media exposure were significantly correlated with a higher risk of under-five mortality. This study highlights that public health programs should enhance health facility delivery, ANC visit, media exposure, maternal education, and adequate birth spacing to decrease the incidence of under-five mortality in East Africa.

## Background

According to the World Health Organization (WHO), under-five mortality is defined as the death of a child before reaching age five [1]. Despite the global progress in under-five mortality [2], East African countries continued to share the huge burden [3]. Reducing under-five mortality is included in the Sustainable Development Goals (SDGs) as an unfinished agenda of MDGs [4]. Despite the substantial decrement in the global under-five mortality rates from 90.6 per 1000 live births in 1990 to 42.5 per 1000 live births in 2015 [5], an estimated 5.3 million children under age five still died annually [6].

The burden of under-five mortality is unevenly distributed across, it is highly concentrated in middle-income and low-income countries [7, 8]. Public health interventions such as Expanded Program of Immunization (EPI) [9, 10], exclusively breastfeeding practice for 6 months [11], and maternal health service utilization [12] such as Antenatal Care (ANC) visit, Postnatal Care (PNC) visit and health facility delivery are the basic strategies implemented by many African countries to reduce under-five mortality [13, 14].

The leading causes of under-five mortality in SSA are diarrheal disease [15], malnutrition [16], pneumonia [17], malaria [18], prematurity [19], and neonatal sepsis [20] which are preventable and treatable. Previous studies revealed that residence [21, 22], maternal education [23], husband education [24], marital status [25], sex of child [26], ANC visit [27], PNC [28], place of delivery [29], preceding birth interval [30], twin births [13], parity [31], maternal age [32], media exposure [33], wealth status [13], child nutritional status [34], mode of delivery [21, 35, 36] and women decision making autonomy [25] as significant predictors of under-five mortality.

Though there are studies reported in several East African countries [29, 37–42], they are unable to capture the incidence and predictors of the level of East African Region. Investigating the inter-country variation in the incidence of under-five mortality and the pooled incidence of under-five mortality at the East Africa level is important for prioritization to design public health interventions. Besides, this study was based on the weighted pooled DHS data of 12 East African countries that have adequate power to detect the true effect of the predictors on under-five mortality using multilevel survival analysis.

## Methods

### Data source

This study was based on the DHS data of 12 East African countries conducted from 2008 to 2019. DHS is a community-based cross-sectional study conducted every five-year to generate updated health and health-related indicators. The DHS conducted in Burundi, Ethiopia, Comoros, Uganda, Rwanda, Tanzania, Mozambique, Madagascar, Zimbabwe, Kenya, Zambia, and Malawi were appended together to determine the incidence and predictors of under-five mortality in East Africa. Each country’s survey consists of different datasets including men, women, children, birth, and household datasets; for this study, we used the Kids Record (KR) file. In the KR file, all mothers with under-five children born in the last 5 years preceding the survey were included. In DHS, a two-stage stratified cluster sampling technique was employed using the Population and Housing Census (PHC) as a sampling frame. In the first stage, Enumeration Areas (EAs) were chosen with probability sampling proportional to the size of the EAs with independent selection in each sampling stratum. In the second stage, households were systematically selected. The detailed sampling procedure was presented in the full DHS report. A total weighted sample of 138,803 live births was included in the study.

### Variables of the study

The dependent variable was under-five mortality. A child who has died within 5 years of birth was considered as an event and those who were alive during the study period were considered as censored. Under-5 mortality is defined as the death of live birth within 59 months of life. Age at death was recorded in months. Given the hierarchical nature of DHS data, independent variables considered for this study were from two sources (individual-level and community-level variables). The individual-level independent variables were maternal education, maternal age, husband education, wealth status, media exposure, marital status, maternal occupation, child age, sex of child, birth order, birth size, birth outcome, birth size, place of delivery, mode of delivery, women health care decision making autonomy, unwanted pregnancy, number of ANC visit and preceding birth interval. The community-level variables were the place of residence, country, and distance to the health facility.

### Data management and analysis

The data were weighted using sampling weight, primary sampling unit, and strata before any statistical analysis to restore the representativeness of the survey and to account for the sampling design to get reliable statistical estimates. The sampling statisticians determine how many samples are needed in each stratum to get reliable estimates, in DHS, some areas were oversampled, and some areas were under-sampled. So, to get statistics that are representative of the country, the distribution of under-5 children in the sample need to be weighted (mathematically adjusted) such that it resembles the true distribution in East Africa by using sampling weight (v005), primary sampling unit (v021) and strata (v022). The descriptive and summary statistics were conducted using STATA version 14 software. The DHS data has a hierarchical structure, and therefore under-5 children were nested within a cluster/EAs. This violates the traditional regression model assumption, which is the independence of observations and equal variance across clusters. The Proportional Hazard (PH) assumption was assessed using global schoenfeld residual test and it was violated (*p*-value< 0.05), and therefore, the cox-proportional model was not the appropriate model for the data. To check whether there was clustering or not using Variance Partition Coefficient (VPC), it showed that there was clustering that should be considered using multilevel survival models to get a reliable estimate. For model selection; log-likelihood ratio test, deviance (−2LL), Akaike Information Criteria (AIC), and Cox-Snell residual plot were applied. A Cox-Snell residual plot considers the distribution and estimated parameters from the lifetime regression model, and the plot of the Weibull regression model was closer to the bisector than the others. Besides, the multilevel Weibull regression model was the best-fitted model as it had the lowest deviance, AIC, and BIC values.

Weibull regression is the most popular parametric model, as it is highly flexible and has simple hazard and survival functions [43, 44]. It provides estimates of the baseline hazard function. Which is characterized by two parameters: one is the shape parameter denoted by γ and the other is the scale parameter denoted by λ. It is noted that when γ = 1, the hazard rate remains constant over time and the distribution turns exponential. The hazard rate increases when γ > 1 and decreases when γ < 1over the lifetimes. As a result, Weibull distribution could be used to model the survival distribution of a population that has either increasing or decreasing or constant risk. Besides, we have checked the distribution of the data follows a Weibull distribution or not by plotting the logarithm of survival function against times, and the logarithm of the survival function decreases sharply from 0 to as time increases. Therefore, variables with a *p*-value less than 0.20 in the bivariable multilevel Weibull regression were included in the multivariable analysis. In the multivariable analysis, the Adjusted Hazard Ratio (AHR) with 95% Confidence Interval (CI) was used to declare significant predictors of under-five mortality.

### Ethical consideration

Permission for data access was obtained from the measure DHS program through an online request from http://www.dhsprogram.com. The data used for this study were publicly available with no personal identifier.

## Result

### Socio-demographic and economic characteristics of study participants

Of the total 138,803 under-five children, about 19,563 (14.1%) were from Kenya, and 3235 (2.3%) were from Comoros. More than three-fourths (78.3%) of children’s mothers were rural residents. About 66,070 (47.6%) of their mother were aged 25–34 years and 73,811 (53.2%) of the mothers attained primary education (Table 1).

**Table 1:** The socio-demographic and economic characteristics of the study participants in East Africa

Variable	Weighted frequency (***N*** = 138,803)	Percentage (%)
**Country**		
Burundi	13,611	9.8
Ethiopia	11,022	7.9
Kenya	19,563	14.1
Comoros	3235	2.3
Madagascar	12,686	9.1
Malawi	17,395	12.5
Mozambique	11,704	8.4
Rwanda	8003	5.8
Tanzania	10,052	7.2
Uganda	15,270	11.0
Zambia	9841	7.1
Zimbabwe	6418	4.6
**Residence**		
Rural	30,108	21.7
Urban	108,695	78.3
**Maternal age**		
15–24	41,683	30.0
25–34	66,070	47.6
≥ 35	31,050	22.4
**Maternal education status**		
No	33,448	24.1
Primary	73,811	53.2
Secondary and above	31,544	22.7
**Husband education status**		
No	22,654	16.3
Primary	57,352	41.3
Secondary and above	58,797	42.4
**Wealth status**		
Poorest	33,229	23.9
Poorer	29,866	21.5
Middle	26,820	19.3
Richer	25,590	18.4
Richest	23,298	16.8
**Media exposure**		
No	48,776	35.1
Yes	90,027	64.9
**Marital status**		
Single	6482	4.7
Married	118,613	85.4
Divorced/widowed/separated	13,708	9.9
**Respondent working**		
No	44,616	32.1
Yes	94,187	67.9

### Maternal obstetric and child-related characteristics

The majority (56.5%) of the children were aged 12–36 months and about 50.6% were females. Nearly three-fourths (72.6%) of the children were born at the health facility and 4415 (3.2%) were multiple births (Table 2).

**Table 2:** The maternal obstetric and child related characteristics of the study participants in East Africa

Variables	Weighted frequency	Percentage (%)
**Child age (in months)**		
< 12	27,400	19.7
12–36	78,409	56.5
37–60	32,994	23.8
**Sex of child**		
Male	70,206	50.6
Female	68,597	49.4
**Birth outcome**		
Single	134,388	96.8
Multiple	4415	3.2
**Birth size**		
Large	40,812	29.4
Average	64,315	46.3
Small	33,676	24.3
**Birth order**		
First	32,654	23.5
2–4	66,883	48.2
≥ 5	39,266	28.3
**Place of delivery**		
Home	38,005	27.4
Health facility	100,798	72.6
**Mode of delivery**		
Vaginal	130,518	94.0
Caesarean delivery	8285	6.0
**Women health care decision making autonomy**		
Respondent alone	84,970	18.6
Jointly with their husband/parent	55,653	40.1
Husband/parent alone	57,402	41.3
**Distance to health facility**		
A big problem	84,970	61.2
Not a big problem	53,833	38.8
**Unwanted pregnancy**		
No	82,691	59.6
Yes	56,112	40.4
**Number of ANC visit**		
No	6127	4.4
1–3	39,791	28.7
≥ 4	92,885	66.9
**Preceding birth interval (in months)**		
< 24	18,963	13.7
24–48	58,591	42.2
≥ 49	61,249	44.1
**Weight/age**		
Normal	126,731	91.3
Moderately underweight	9089	6.6
Severely underweight	2983	2.2
**Height/age**		
Normal	107,654	77.6
Moderately stunted	19,032	13.7
Severely stunted	12,117	8.7
**Weight/height**		
Normal	134,523	96.9
Moderately wasted	3044	2.2
Severely wasted	1236	0.9

### Incidence of under-five mortality

**Fig. 1:**
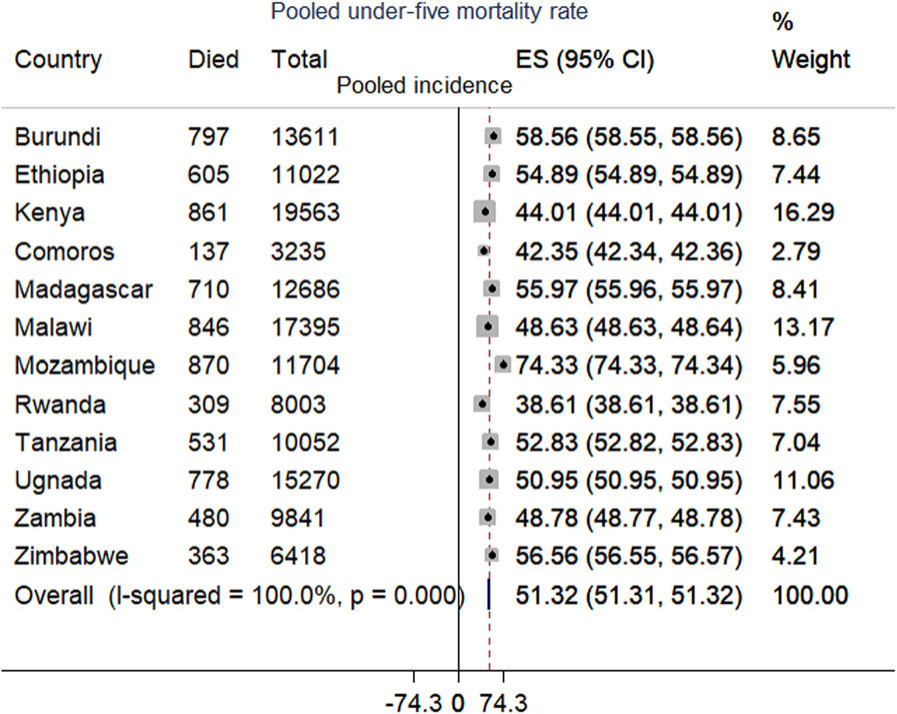
The forest plot of the pooled incidence of under-five mortality in and across East African countries

**Fig. 2:**
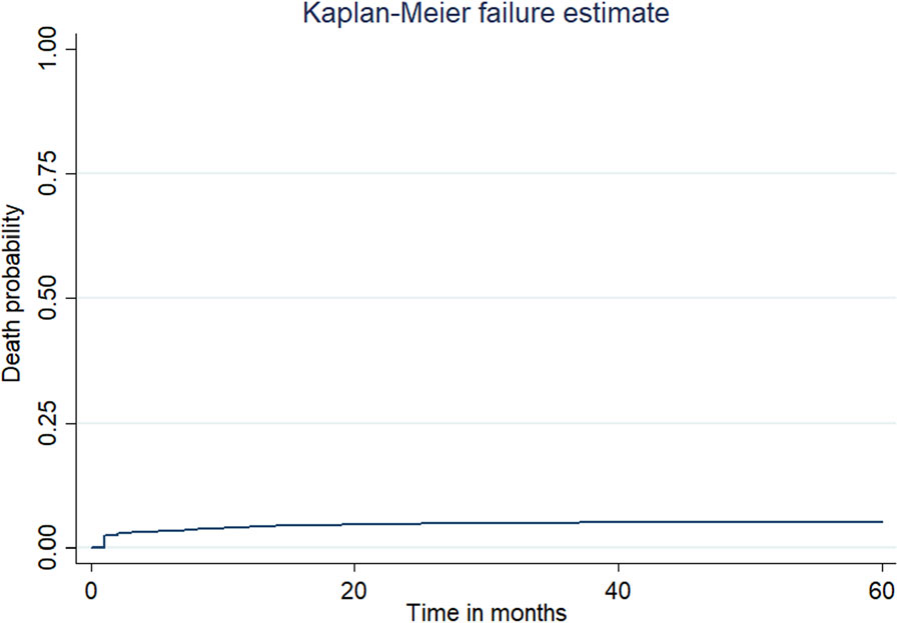
The overall Kaplan-Meier curve of the survival status of under-5 mortality in East Africa

The under-five mortality rate in East Africa was 51.318 (95% CI: 51.311, 51.323) per 1000 live births, ranged from 38.608 (95% CI: 38.605, 38.615) per 1000 live births in Rwanda to 74.333 (95% CI: 74.332, 74.341) to 1000 live births in Mozambique (Fig. 1). The overall Kaplan-Meier failure curve indicated that the probability of under-five mortality increased over time. The risk of dying was increased alarmingly in the first year of life and slowly to aged 3 years and then it remains steady (Fig. 2). The log-rank test showed that there was a significant difference in under-five mortality probability across the residence, country, mode of delivery, birth order, ANC, birth outcome, place of delivery, preceding birth interval, health insurance coverage, distance to the health facility, wanted pregnancy, birth size, maternal education, husband education, respondent age, twin pregnancy, and wealth index (log-rank, *p* <  005) (Table 3). The PH assumption was violated (Global Schoenfeld residual test, *p* <  0.0001), indicated that the Cox-proportional model was not an appropriate model (Table 4). Therefore, parametric models were fitted and the Weibull regression model was the best-fitted model (Table 5). Besides, in the null model, the VPC was 0.11, which showed that about 11% of the total variability in under-five mortality was due to clustering. In addition, the LR-test was significant it indicates the multilevel Weibull regression model was the best-fitted model for the data. We have fitted four models and the final model was the best-fitted model since it had the lowest deviance (Table 6).

**Table 3:** Log rank test for the predictors of under-five mortality in East Africa

Variable	*p*-value	Variable	*p*-value
Residence	< 0.0001	Mode of delivery	0.03
Country	< 0.0001	Media exposure	0.14
Covered by health insurance	< 0.0001	Maternal education	< 0.0001
Wealth index	< 0.0001	Husband education	< 0.0001
Birth outcome	< 0.0001	Wanted pregnancy	< 0.0001
Distance to health facility	< 0001	Women decision making autonomy	0.005
Place of delivery	< 0.0001	Maternal age	< 0.0001
ANC visit during pregnancy	< 0.0001	Birth order	< 0.0001
Birth interval	< 0.0001	Birth size	< 0.001
Sex of child	< 0.001

**Table 4:** Schoenfeld residual test for checking proportional hazard assumption for the incidence of under-five mortality and its predictors among live births in the 12 East African countries

Variables	Rho	Chi2	Df	Prob > chi2
Residence	0.006	0.23	1	0.62
Country	−0.022	3.54	1	0.06
Wealth index	−0.04	12.48	1	0.0004
Sex of child	0.045	16.53	1	< 0.001
Women education	−0.02	2.95	1	0.09
Husband education	−0.034	8.53	1	0.004
Place of delivery	−0.022	3.56	1	0.06
ANC visit	0.084	58.48	1	< 0.001
Birth interval	−0.006	0.23	1	0.63
Mode of delivery	−0.03	5.54	1	0.02
Birth outcome	−0.08	42.99	1	< 0.001
Maternal age	0.04	13.76	1	0.0002
Media exposure	−0.011	0.90	1	0.34
Wanted pregnancy	0.018	2.24	1	0.13
Distance to health facility	−0.017	2.04	1	0.15
Covered by health insurance	0.0006	0.001	1	0.96
Women autonomy	0.0007	0.001	1	0.95
Maternal age	0.003	0.07	1	0.79
Birth order	−0.0019	0.03	1	0.987
Birth size	−0.093	71.41	1	< 0.001
Global test		333.84	20	< 0.0001

**Table 5:** Model comparison parameters

Parameter	Deviance	AIC	BIC
Weibull regression	84,122.42	84,164.42	84,371.03
exponential regression	112,215.08	112,255.1	112,451.9
gamma regression	99,804.58	99,846.59	100,053.2
lognormal regression	99,575.74	99,617.74	99,824.35
log- logistic regression	98,813.36	98,855.35	99,061.96

**Table 6:** The multilevel Weibull regression analysis of individual and community level predictors of under-five mortality in East Africa

Variable	Null model	Model 1 (AHR with 95% CI)	Model 2(AHR with 95% CI)	Model 3 (AHR with 95% CI)
**Maternal age**				
15–24		1		1
25–34		0.94 (0.88, 1.01)		0.95 (0.89, 1.01)
≥ 35		1.13 (1.03, 1.23)		1.13 (1.04, 1.24)*
**Maternal education status**				
No		1		1
Primary		1.04 (0.98, 1.11)		1.06 (0.99, 1.13)
Secondary and above		0.81 (0.74, 0.88)		0.83 (0.75, 0.91)*
**Husband education status**				
No		1		1
Primary		1.03 (0.96, 1.11)		1.03 (0.96, 1.11)
Secondary and above		1.00 (0.92, 1.08)		1.03 (0.95, 1.12)
**Media exposure**				
Yes		1		1
No		1.10 (1.05, 1.17)		1.13 (1.07, 1.20)*
**Birth outcome**				
Single		1		1
Multiple		3.87 (3.57, 4.18)		3.81 (3.52, 4.12)*
**Sex of child**				
Male		1.23 (1.18, 1.29)		1.27 (1.21, 1.33)*
Female		1		1
**Birth size**				
Average		1		1
small		1.69 (1.60, 1.79)		1.73 (1.63, 1.84)*
Large		1.15 (1.08, 1.22)		1.11 (1.04, 1.17)*
**Birth order**				
First		1		1
2–4		0.61 (0.56, 0.67)		0.62 (0.56, 0.67)*
≥ 5		0.68 (0.61, 0.76)		0.68 (0.61, 0.76)*
**Place of delivery**				
Home		1		1
Health facility		0.95 (0.90, 1.01)		0.87 (0.82, 0.93)*
**Mode of delivery**				
Vaginal		1		1
Caesarean section		1.06 (0.96, 1.17)		1.09 (0.99, 1.21)
**Number of ANC visit**				
No visit		1		1
1–3		0.61 (0.54, 0.68)		0.61 (0.54, 0.68)*
≥ 4		1.01 (0.90, 1.13)		1.02 (0.91, 1.14)
**Preceding birth interval (in months)**				
< 24		1		1
24–48		0.54 (0.51, 0.58)		0.53 (0.50, 0.57)*
≥ 49		0.50 (0.46, 0.54)		1.02 (0.91, 1.14)
**Wanted birth**				
No		1		1
Yes		0.68 (0.65, 0.72)		0.72 (0.68, 0.76)*
**Wealth status**				
Poorest		1		1
Poorer		0.98 (0.91, 1.04)		0.95 (0.89, 1.02)
Middle		0.93 (0.86, 1.01)		0.90 (0.83, 0.97)*
Richer		0.99 (0.91, 1.06)		0.93 (0.86, 1.01)
Richest		0.89 (0.82, 0.98)		0.81 (0.73, 0.90)*
**Women health care decision making autonomy**				
Respondent alone		1		1
Jointly with husband/partner		1.07 (1.01, 1.15)		1.06 (0.99, 1.14)
Husband/partner alone		1.13 (1.06, 1.21)		1.15 (1.07, 1.23)*
**Residence**				
Rural			1.12 (1.05, 1.18)	0.95 (0.89, 1.03)
Urban			1	1
**Country**				
Rwanda			1	1
Burundi			1.43 (1.25, 1.64)	1.23 (1.06, 1.44)*
Ethiopia			1.57 (1.37, 1.81)	1.01 (0.84, 1.19)
Kenya			1.12 (0.98, 1.28)	0.79 (0.68, 0.91)*
Comoros			1.08 (0.87, 1.33)	0.66 (0.53, 0.83)*
Madagascar			1.49 (1.30, 1.71)	1.10 (0.95, 1.28)*
Malawi			1.25 (1.09, 1.49)	1.07 (0.91, 1.25)
Mozambique			1.97 (1.73, 2.26)	1.42 (1.21, 1.66)*
Tanzania			1.35 (1.17, 1.55)	1.01 (0.86, 1.20)
Uganda			1.38 (1.21, 1.57)	0.98 (0.84, 1.16)
Zambia			1.27 (1.11, 1.47)	1.02 (0.86, 1.22)
Zimbabwe			1.43 (1.22, 1.68)	1.20 (1.01, 1.45)*
**Distance to health facility**				
Not a big problem			1	1
A big problem			1.03 (0.98, 1.09)	0.98 (0.93, 1.03)
VPC	0.11	0.08	0.09	0.07
LR test	LR test vs. Weibull model: chibar2(01) = 27.11 Prob > = chibar2 = 0.0000	LR test vs. Weibull model: chibar2(01) = 20.46 Prob > = chibar2 = 0.0000	LR test vs. Weibull model: chibar2(01) = 22.93 Prob > = chibar2 = 0.0000	LR test vs. Weibull model: chibar2(01) = 17.87 Prob > = chibar2 = 0.0000
Deviance	85,876.04	83,370.89	85,648.85	83,211.55

* *P*-value < 0.05, ** *p*-value < 0.01, *ANC* Antenatal Care, *VPC* Variance Partition Coefficient, *LR* Likelihood Ratio

### Predictors of under-five mortality

Maternal age, maternal education, media exposure, birth outcome, sex of the child, birth size, birth order, place of delivery, number of ANC visits, preceding birth interval, wanted pregnancy, wealth, women autonomy and country were significant predictors of under-five mortality. Children born to mothers aged 35 years and above had 1.13 times (AHR = 1.13, 95% CI: 1.04, 1.24) higher hazard of death in the first 5 years compared to a child born to a mother aged 15–24 years. A child born to a mother who attained secondary education and above had decreased risk of under-five mortality by 17% (AHR = 0.83, 95% CI: 0.75, 0.91) than a child born to a mother who had no formal education. A child born to a mother who had no media exposure was 1.13 times (AHR = 1.13, 95%: 1.07, 1.20) higher risk of under-five mortality than a child born to a mother who had media exposure. The hazard of under-five mortality among twin births was 3.81 times (AHR = 3.81, 95% CI: 3.52, 4.12) higher than single births. Being male had an increased hazard of under-five mortality by 27% (AHR = 1.27, 95% CI: 1.21, 1.33) than a female child. Children who were small and large size at birth had 1.73 times (AHR = 1.73, 95% CI: 1.63, 1.84) and 1.11 times (AHR = 1.11, 95% CI: 1.04, 1.17) higher hazard of under-five mortality than average size baby at birth respectively.

The hazard of under-five mortality among second to fourth, and five and above birth order was decreased by 38% (AHR = 0.62, 95% CI: 0.56, 0.67), and 32% (AHR = 0.68, 95% CI: 0.61, 0.76) than first birth respectively. The hazard of under-five mortality among children born at a health facility was decreased by 13% (AHR = 0.87, 95% CI: 0.82, 0.93) than those born at home. Children born to mothers who had 1–3 ANC visits during pregnancy had 0.61 times decreased hazard of mortality (AHR = 0.61, 95% CI: 0.54, 0.68) than a child born to a mother who didn’t have ANC visit during pregnancy. Children who were born within 24–48 birth intervals had decreased hazard of mortality by 47% (AHR = 0.53, 95% CI: 0.50, 0.57) than a child born within 23 months of birth interval. Children who were wanted having decreased hazard of mortality within 5 years of birth by 28% (AHR = 0.72, 95% CI: 0.68, 0.76) than unwanted childbirth.

The hazard of under-five mortality among child in the middle and richest household were decreased by 10% (AHR = 0.90, 95% CI: 0.83, 0.97) and 19% (AHR = 0.81, 95% CI: 0.73, 0.90) than child in the poorest household, respectively. Child in Burundi, Kenya, Comoros, Madagascar, Mozambique, and Zimbabwe were 1.23 times (AHR = 1.23, 95% CI: 1.06, 1.44), 0.79 times (AHR = 0.79, 95% CI: 0.68, 0.91), 0.66 times (AHR = 0.66, 95% CI: 0.53, 0.83), 1.10 times (AHR = 1.10, 95% CI: 0.95, 1.28), 1.42 times (AHR = 1.42, 95% CI: 1.21, 1.66), and 1.20 times (AHR = 1.20, 95% CI: 1.01, 1.45) higher hazard of under-five mortality than child in Rwanda respectively (Table 6).

## Discussion

Under-5 mortality is an important global public health issue particularly in Sub-Saharan Africa since it is commonly used as one of the most sensitive measures of countries’ well-being and development. Newborns and children have improved substantially over the last three decades even though the targeted two-thirds reduction was not achieved in East Africa. The incidence of neonatal mortality rate in East Africa was 51.32 (95% CI: 51.31, 51.32) per 1000 live births, and significantly varied across the East African countries. It was higher than the SDG target for child mortality to reduce under-5 mortality of at least as low as 25 deaths per 1000 live births [45]. It could be due to lack of access to healthcare facilities, improper hygiene and sanitation, unclean water and continued risk of malnutrition in East African countries contributes to the huge burden of under-five mortality [46]. Besides, many of the African countries are continued to be prone to the leading causes of infectious diseases such as pneumonia, malaria, and diarrheal diseases that are closely linked to poverty [47, 48].

In this study maternal age, maternal education, media exposure, birth outcome, sex of the child, birth size, birth order, place of delivery, number of ANC visits, preceding birth interval, wanted pregnancy, wealth status, and women autonomy were significant predictors of under-five mortality. Maternal age was the important predictor of under-5 mortality in East Africa, children born to mothers aged 35 years and above had a higher risk of under-5 mortality than children born to mothers aged 15–24 years. It was consistent with studies reported in Nigeria [49], and SSA [21, 50], it could be due to children born to advance age mothers are at higher risk of low birth weight, prematurity, a congenital abnormality such as down syndrome, and malnutrition, this might be the possible reason for the increased risk of under-5 mortality among births to advanced age mothers [51]. Maternal education and media exposure were significant predictors of under-5 mortality. It was in line with study findings in SSA [23], India [52], Nigeria [33], and Ghana [13], this might be because maternal education and media exposure are the most powerful tool in reducing under-five mortality [53]. Educated mothers and mothers who have media exposure are more likely to use information more effectively when caring for children and tend to seek appropriate health care more effectively than mothers who are not educated [54, 55]. Besides, educated mothers are more aware of the importance of childhood vaccination and basic child health care services. This could reduce the risk of under-five mortality [56]. Health facility delivery and ANC visit were significant predictors of decreased risk of under-5 mortality. It was consistent with studies reported in Nigeria [57], and SSA [21, 58], it is since ANC visit during pregnancy and health facility delivery has a direct effect through prevention of infection, birth trauma, asphyxia and to rapidly detect and treat the complications [59]. Furthermore, ANC visit and health facility delivery is the entry point for the utilization of basic children health care services such as PNC visit, and childhood vaccination that could prevent the leading causes of under-5 mortality like pneumonia, malaria, and diarrheal diseases. This might be the possible explanation for the decreased risk of under-five mortality [60]. Male children were at increased risk of under-five mortality, this was supported by previous studies reported in India [61] and Iraq [62]. The excess risk of mortality among male children has been because the male sex is more vulnerable to morbidities such as low Apgar score, Intra-uterine Growth Restriction (IUGR), respiratory insufficiency, and prematurity than female sex [63]. As well, there is a hormonal difference among males and females, that males have a higher level of testosterone that has an association with pulmonary biomechanics and vascular development that could make males more vulnerable to respiratory and neurological diseases [64, 65]. Twin births were at higher risk of under-5 mortality than singletons. This was consistent study findings in Ethiopia [66], Burkina Faso [67], and Jordan [68], the excess risk of under-5 mortality among twin births is due to twin births are more likely to be born prematurely, to have intrauterine growth restriction, to be of lower birth weight, to have congenital abnormalities and to have complications around the time of labor and delivery, such as umbilical cord prolapse or premature separation of the placenta than singletons this might be the possible justifications [69, 70]. Having a preceding birth interval of 24–48 months had decreased the hazard of under-5 mortality than less than 24 months of birth interval. This was consistent with prior findings reported in developing countries [30, 71], the possible explanation could be due to the reason that mothers having shorter preceding birth intervals are less able to provide nourishment for the fetus because her body has less time to recuperate from the previous pregnancy. In addition, the uterus had less time to recover, and also lactation will deplete maternal nutrition [72] this could result in low birth weight, child malnutrition, lack of care, attention, and competition of children that could increase the risk of under-5 mortality. Children who were a small size or large size at birth had a higher hazard of under-five mortality than children who were average size at birth, it could be due to low birth weight babies and macrosomic babies have immaturity of organs and underlined medical conditions like congenital heart diseases, down syndrome, HIV/AIDS, and Diabetic Mellitus (DM) that might increase their risk of mortality within 5 years of life. Birth order has a significant influence on the hazard of under-5 mortality. Under-5 mortality was highest in the first birth, it was consistent with studies reported in Nigeria [49] and India [53]. The possible explanation is that the first birth has increased susceptibility to pregnancy-related complications such as Antepartum Haemorrhage (APH), preeclampsia, prematurity, and fetal distress that could increase their risk of mortality before their fifth birthday. Wealth inequality and women’s autonomy in health care decision-making had a significant effect on child survival, it was consistent with Bangladesh [73] and Ghana [74]. Under-5 mortality is highly concentrated in developing countries and is considered an indicator of the country’s poverty level [74, 75]. Children from wealthier households might have good nutrition and childhood health care services such as vaccination service, sunlight exposure, and good breastfeeding practices [76]. Unwanted births had a higher risk of under-five mortality than wanted births, this was supported by previous studies reported in Tanzania [42], it could be due to unwanted births may not have got adequate nutrition and basic childhood services.

This study has several strengths. First, the study was based on pooled weighted nationally representative DHS surveys of 12 East African countries that were weighted to make the resulting representative and to get a reliable estimate. Secondly, multilevel survival analysis was fitted by considering the hierarchical nature of the DHS data to get identify community and individual-level predictors of under-5 mortality. Furthermore, the study was based on the large sample size, this could increase the power of the study to get the true effect of the predictors. This finding should be interpreted in light of the following limitations. First, this study was based upon recall by mothers and it is prone to recall bias. Second, the pooled incidence of under-five mortality has considerable heterogeneity across countries (I^2^ < 0.05), and we tried to identify the source of heterogeneity through meta-regression and sub-group analysis but this was not significant. Furthermore, variables such as underlined medical conditions such as congenital heart diseases, pneumonia, malaria, etc. were not included in this study since these variables were not collected in DHS. Moreover, the DHS survey year was not the same in all countries, it was based on DHS conducted 2008 to 2019. This might overestimate or underestimate the incidence of under-five mortality.

### Policy implications

Globally, evidence on the incidence and predictors of under-five mortality has grown substantially currently. This information has been used as a preventive measure that is linked to maternal and child health. From a policy point of view, the interventions which are designed to tackle under-five mortality such as childhood vaccination, health facility delivery, periodic child growth monitoring, exclusive breastfeeding practice, and maternal education should be scaled to sustain the reduction in under-five mortality in East African countries. Maternal education is central to improve birth spacing, childhood mortality, and morbidity. Enhancing the availability of education to women is needed to increases the chance of child survival as they adhered to the maternal and child health guidelines and recommendations.

## Conclusion

Under-five mortality remains the major public health problem in East Africa which significantly varied across countries. The multilevel survival analysis demonstrated different individual and community level predictors that have a significant influence on under-5 mortality. Advanced maternal age, twin births, low birth weight, macrosomia, and women who didn’t participate in health care decision-making autonomy were significantly associated with an increased hazard of under-5 mortality. Whereas, maternal education, media exposure, higher order of birth, health facility delivery, having ANC visit, birth interval ≥ 24 months, wanted pregnancy and rich wealth index were significantly associated with a lower risk of under-5 mortality. These findings are important to guide public health programs and interventions targeting enhancing health facility delivery, empowering women in making health care decision making, ANC visits, and birth space using family planning services to reduce the incidence of under-5 mortality in East Africa.

## Data Availability

Data is available online and you can access it from *www.measuredhs*.com.

## Abbreviations

AHR: Adjusted hazard ratio
AIC: Akakie information criteria
ANC: Antenatal care
AHR: Adjusted hazard ratio
CI: Confidence interval
DHS: Demographic health survey
DM: Diabetic mellitus
LLR: Log-likelihood ratio
PH: Proportional hazard
PNC: Postnatal care
SDGs: Sustainable development goals
SSA: Sub-Saharan Africa
VPC: Variance partition coefficient
WHO: World Health Organization

## References

[1] Ahmad OB, Lopez AD, Inoue M (2000). The decline in child mortality: a reappraisal. Bull World Health Organ.

[2] Lim SS, Allen K, Bhutta ZA, Dandona L, Forouzanfar MH, Fullman N, Gething PW, Goldberg EM, Hay SI, Holmberg M (2016). Measuring the health-related sustainable development goals in 188 countries: a baseline analysis from the global burden of disease study 2015. Lancet.

[3] Lozano R, Fullman N, Abate D, Abay SM, Abbafati C, Abbasi N, Abbastabar H, Abd-Allah F, Abdela J, Abdelalim A (2018). Measuring progress from 1990 to 2017 and projecting attainment to 2030 of the health-related sustainable development goals for 195 countries and territories: a systematic analysis for the global burden of disease study 2017. Lancet.

[4] Kumar S, Kumar N, Vivekadhish S (2016). Millennium development goals (MDGS) to sustainable development goals (SDGS): addressing unfinished agenda and strengthening sustainable development and partnership. Indian J Commun Med.

[5] Khodaee GH, Khademi G, Saeidi M (2015). Under-five Mortality in the World (1900–2015). Int J Pediatr.

[6] Liu L, Oza S, Hogan D, Chu Y, Perin J, Zhu J, Lawn JE, Cousens S, Mathers C, Black RE (2016). Global, regional, and national causes of under-5 mortality in 2000–15: an updated systematic analysis with implications for the sustainable development goals. Lancet.

[7] Kazembe L, Clarke A, Kandala N-B: Childhood mortality in sub-Saharan Africa: cross-sectional insight into small-scale geographical inequalities from Census data. BMJ Open 2012, **2**(5), 2, 5, 10.1136/bmjopen-2012-001421. 10.1136/bmjopen-2012-001421 PMC3488715 23089207

[8] Wang H, Liddell CA, Coates MM, Mooney MD, Levitz CE, Schumacher AE, Apfel H, Iannarone M, Phillips B, Lofgren KT (2014). Global, regional, and national levels of neonatal, infant, and under-5 mortality during 1990–2013: a systematic analysis for the global burden of disease study 2013. Lancet.

[9] Ngowu R, Larson JS, Kim MS (2008). Reducing child mortality in Nigeria: a case study of immunization and systemic factors. Soc Sci Med.

[10] Duclos P, Okwo-Bele J-M, Gacic-Dobo M, Cherian T (2009). Global immunization: status, progress, challenges and future. BMC Int Health Hum Rights.

[11] Azuine RE, Murray J, Alsafi N, Singh GK (2015). Exclusive breastfeeding and under-five mortality, 2006-2014: a cross-national analysis of 57 low-and-middle income countries. Int J MCH AIDS.

[12] Rutherford ME, Mulholland K, Hill PC (2010). How access to health care relates to under-five mortality in sub-Saharan Africa: systematic review. Tropical Med Int Health.

[13] Aheto JMK (2019). Predictive model and determinants of under-five child mortality: evidence from the 2014 Ghana demographic and health survey. BMC Public Health.

[14] Seid AM, Yesuf ME, Koye DN (2013). Prevalence of exclusive breastfeeding practices and associated factors among mothers in Bahir Dar city, Northwest Ethiopia: a community based cross-sectional study. Int Breastfeed J.

[15] Nyamwaya BM. To establish the causes of under-five mortality at Kampala International University teaching hospital: Kampala International University, College of Medicine and Surgery; 2017.

[16] Mose BN (2013). Causes of under-five mortality at Kampala International University teaching hospital.

[17] Mekonnen W, Assefa N, Asnake W, Sahile Z, Hailemariam D. Under five causes of death in Ethiopia between 1990 and 2016**:** Systematic review with meta-analysis. Ethiop J Health Dev (EJHD). 2020;34(2).

[18] Adewemimo A, Kalter HD, Perin J, Koffi AK, Quinley J, Black RE (2017). Direct estimates of cause-specific mortality fractions and rates of under-five deaths in the northern and southern regions of Nigeria by verbal autopsy interview. PLoS ONE.

[19] Xu Y-H, Huang X-W, Yang R-L (2011). The under-five mortality rate and the causes of death in Zhejiang Province between 2000 and 2009. Zhongguo Dang Dai Er Ke Za Zhi.

[20] Ntuli ST, Malangu N, Alberts M (2013). Causes of deaths in children under-five years old at a tertiary hospital in Limpopo province of South Africa. Global J Health Sci.

[21] Yaya S, Bishwajit G, Okonofua F, Uthman OA (2018). Under five mortality patterns and associated maternal risk factors in sub-Saharan Africa: a multi-country analysis. PLoS ONE.

[22] Sastry N (2004). Urbanization, development and under-five mortality differentials by place of residence in São Paulo, Brazil, 1970-1991. Demogr Res.

[23] Monden CW, Smits J (2013). Maternal education is associated with reduced female disadvantages in under-five mortality in sub-Saharan Africa and southern Asia. Int J Epidemiol.

[24] Aly HY, Grabowski R (1990). Education and child mortality in Egypt. World Dev.

[25] Kanmiki EW, Bawah AA, Agorinya I, Achana FS, Awoonor-Williams JK, Oduro AR, Phillips JF, Akazili J (2014). Socio-economic and demographic determinants of under-five mortality in rural northern Ghana. BMC Int Health Hum Rights.

[26] Costa JC, ICM d S, Victora CG. Gender bias in under-five mortality in low/middle-income countries. BMJ Global Health. 2017;2(2). 10.1136/bmjgh-2017-000350 PMC5656133 29082002

[27] Machio PM (2018). Determinants of neonatal and under-five mortality in Kenya: do antenatal and skilled delivery care services matter?. J Afr Dev.

[28] Bosomprah S, Ragno PL, Gros C, Banskota H (2015). Health insurance and maternal, newborn services utilisation and under-five mortality. Arch Public Health.

[29] Ettarh R, Kimani J (2012). Determinants of under-five mortality in rural and urban Kenya. Rural Remote Health.

[30] Rutstein SO (2005). Effects of preceding birth intervals on neonatal, infant and under-five years mortality and nutritional status in developing countries: evidence from the demographic and health surveys. Int J Gynecol Obstet.

[31] Kozuki N, Sonneveldt E, Walker N (2013). Residual confounding explains the association between high parity and child mortality. BMC Public Health.

[32] Sinha S, Aggarwal AR, Osmond C, Fall CH, Bhargava SK, Sachdev HS (2016). Maternal age at childbirth and perinatal and under-five mortality in a prospective birth cohort from Delhi. Indian Pediatr.

[33] Adebowale SA, Morakinyo OM, Ana GR (2017). Housing materials as predictors of under-five mortality in Nigeria: evidence from 2013 demographic and health survey. BMC Pediatr.

[34] Munthali T, Jacobs C, Sitali L, Dambe R, Michelo C (2015). Mortality and morbidity patterns in under-five children with severe acute malnutrition (SAM) in Zambia: a five-year retrospective review of hospital-based records (2009–2013). Arch Public Health.

[35] Wegbom AI, Essi ID, Kiri VA (2019). Survival analysis of under-five mortality and its associated determinants in Nigeria: evidence from a survey data. Int J Stat Appl.

[36] Mani K, Dwivedi SN, Pandey RM (2012). Determinants of under-five mortality in rural empowered action group states in India: an application of cox frailty model. Int J MCH AIDS.

[37] Deribew A, Tessema F, Girma B (2007). Determinants of under-five mortality in Gilgel gibe field research center, Southwest Ethiopia. Ethiop J Health Dev.

[38] Gebretsadik S, Gabreyohannes E. Determinants of under-five mortality in high mortality regions of Ethiopia: an analysis of the 2011 Ethiopia demographic and health survey data. Int J Popul Res. 2016:2016–7. 10.1155/2016/1602761.

[39] Diouf K, Tabatabai P, Rudolph J, Marx M (2014). Diarrhoea prevalence in children under five years of age in rural Burundi: an assessment of social and behavioural factors at the household level. Glob Health Action.

[40] Hategeka C, Tuyisenge G, Bayingana C, Tuyisenge L (2019). Effects of scaling up various community-level interventions on child mortality in Burundi, Kenya, Rwanda, Uganda and Tanzania: a modeling study. Global Health Res Policy.

[41] Mturi AJ, Curtis SL (1995). The determinants of infant and child mortality in Tanzania. Health Policy Plan.

[42] Ogbo FA, Ezeh OK, Awosemo AO, Ifegwu IK, Tan L, Jessa E, Charwe D, Agho KE (2019). Determinants of trends in neonatal, post-neonatal, infant, child and under-five mortalities in Tanzania from 2004 to 2016. BMC Public Health.

[43] Royston P (2001). Flexible parametric alternatives to the cox model, and more. Stata J.

[44] Bebbington M, Lai C-D, Zitikis R (2007). A flexible Weibull extension. Reliab Eng Syst Saf.

[45] You D, Hug L, Ejdemyr S, Idele P, Hogan D, Mathers C, Gerland P, New JR, Alkema L (2015). Global, regional, and national levels and trends in under-5 mortality between 1990 and 2015, with scenario-based projections to 2030: a systematic analysis by the UN inter-agency Group for Child Mortality Estimation. Lancet.

[46] Prüss-Ustün A, Wolf J, Bartram J, Clasen T, Cumming O, Freeman MC, Gordon B, Hunter PR, Medlicott K, Johnston R (2019). Burden of disease from inadequate water, sanitation and hygiene for selected adverse health outcomes: an updated analysis with a focus on low-and middle-income countries. Int J Hyg Environ Health.

[47] Kent MM, Yin S. Controlling infectious diseases. Popul Bulletin-Washington. 2006;61(2).

[48] Walker CLF, Rudan I, Liu L, Nair H, Theodoratou E, Bhutta ZA, O'Brien KL, Campbell H, Black RE (2013). Global burden of childhood pneumonia and diarrhoea. Lancet.

[49] Kayode GA, Adekanmbi VT, Uthman OA (2012). Risk factors and a predictive model for under-five mortality in Nigeria: evidence from Nigeria demographic and health survey. BMC Pregnancy Childbirth.

[50] Yaya S, Uthman OA, Okonofua F, Bishwajit G (2019). Decomposing the rural-urban gap in the factors of under-five mortality in sub-Saharan Africa? Evidence from 35 countries. BMC Public Health.

[51] Lozano R, Naghavi M, Foreman K, Lim S, Shibuya K, Aboyans V, Abraham J, Adair T, Aggarwal R, Ahn SY (2012). Global and regional mortality from 235 causes of death for 20 age groups in 1990 and 2010: a systematic analysis for the global burden of disease study 2010. Lancet.

[52] Mandal S, Paul P, Chouhan P. Impact of maternal education on under-five mortality of children in India: insights from the National Family Health Survey, 2005–2006 and 2015–2016. Death Stud. 2019;(10):1–7. 10.1080/07481187.2019.1692970. 10.1080/07481187.2019.1692970 31746268

[53] Singh R, Tripathi V (2013). Maternal factors contributing to under-five mortality at birth order 1 to 5 in India: a comprehensive multivariate study. Springerplus.

[54] Andriano L, Monden CW (2019). The causal effect of maternal education on child mortality: evidence from a quasi-experiment in Malawi and Uganda. Demography.

[55] Muhuri PK (1995). Health programs, maternal education, and differential child mortality in Matlab, Bangladesh. Popul Dev Rev.

[56] Ibnouf A, Van den Borne H, Maarse J (2007). Factors influencing immunisation coverage among children under five years of age in Khartoum State, Sudan. S Afr Fam Pract.

[57] Ezeh OK, Agho KE, Dibley MJ, Hall JJ, Page AN (2015). Risk factors for postneonatal, infant, child and under-5 mortality in Nigeria: a pooled cross-sectional analysis. BMJ Open.

[58] Doctor HV, Nkhana-Salimu S, Abdulsalam-Anibilowo M (2018). Health facility delivery in sub-Saharan Africa: successes, challenges, and implications for the 2030 development agenda. BMC Public Health.

[59] Lawn J, Kerber K, Enweronu-Laryea C, Massee Bateman O (2009). Newborn survival in low resource settings—are we delivering?. BJOG Int J Obstet Gynaecol.

[60] Gilmore B, McAuliffe E (2013). Effectiveness of community health workers delivering preventive interventions for maternal and child health in low-and middle-income countries: a systematic review. BMC Public Health.

[61] Sahu D, Nair S, Singh L, Gulati B, Pandey A (2015). Levels, trends & predictors of infant & child mortality among scheduled tribes in rural India. Indian J Med Res.

[62] Awqati NA, Ali MM, Al-Ward NJ, Majeed FA, Salman K, Al-Alak M, Al-Gasseer N (2009). Causes and differentials of childhood mortality in Iraq. BMC Pediatr.

[63] Ergaz Z, Avgil M, Ornoy A (2005). Intrauterine growth restriction—etiology and consequences: what do we know about the human situation and experimental animal models?. Reprod Toxicol.

[64] Orshal JM, Khalil RA (2004). Gender, sex hormones, and vascular tone. Am J Phys Regul Integr Comp Phys.

[65] Zhao D, Zou L, Lei X, Zhang Y (2017). Gender differences in infant mortality and neonatal morbidity in mixed-gender twins. Sci Rep.

[66] Dejene T, Girma E (2013). Social determinants of under-five mortality in Ethiopia: event history analysis using evidence from Ethiopian demographic and health survey (EDHS).

[67] Becher H, Müller O, Jahn A, Gbangou A, Kynast-Wolf G, Kouyaté B (2004). Risk factors of infant and child mortality in rural Burkina Faso. Bull World Health Organ.

[68] Islam MM, Marium U (2019). Twin births in Jordan: incidence, trends, risk factors and implications for under-five mortality: evidence from the 2012 Jordan population and family health survey. J Biosoc Sci.

[69] Chauhan SP, Scardo JA, Hayes E, Abuhamad AZ, Berghella V (2010). Twins: prevalence, problems, and preterm births. Am J Obstet Gynecol.

[70] Moise J, Laor A, Armon Y, Gur I, Gale R (1998). The outcome of twin pregnancies after IVF. Hum Reprod (Oxford, England).

[71] Kozuki N, Walker N (2013). Exploring the association between short/long preceding birth intervals and child mortality: using reference birth interval children of the same mother as comparison. BMC Public Health.

[72] DaVanzo J, Hale L, Razzaque A, Rahman M (2007). Effects of interpregnancy interval and outcome of the preceding pregnancy on pregnancy outcomes in Matlab, Bangladesh. BJOG Int J Obstet Gynaecol.

[73] Yaya S, Bishwajit G (2019). Burden of acute respiratory infections among under-five children in relation to household wealth and socioeconomic status in Bangladesh. Trop Med Infect Dis.

[74] Kumi-Kyereme A, Amo-Adjei J (2016). Household wealth, residential status and the incidence of diarrhoea among children under-five years in Ghana. J Epidemiol Global Health.

[75] Wagstaff A, Watanabe N (1999). Socioeconomic inequalities in child malnutrition in the developing world: the World Bank.

[76] Black RE, Allen LH, Bhutta ZA, Caulfield LE, De Onis M, Ezzati M, Mathers C, Rivera J (2008). Maternal, group CUS: maternal and child undernutrition: global and regional exposures and health consequences. Lancet.

